# Spatial clustering and contextual factors associated with hospitalisation and deaths due to COVID-19 in Sweden: a geospatial nationwide ecological study

**DOI:** 10.1136/bmjgh-2021-006247

**Published:** 2021-07-28

**Authors:** Osvaldo Fonseca-Rodríguez, Per E Gustafsson, Miguel San Sebastián, Anne-Marie Fors Connolly

**Affiliations:** 1 Department of Clinical Microbiology, Umeå University, Umeå, Sweden; 2 Department of Epidemiology and Global Health, Umeå University, Umeå, Sweden

**Keywords:** COVID-19, epidemiology, public health, other study design

## Abstract

**Introduction:**

In Sweden, thousands of hospitalisations and deaths due to COVID-19 were reported since the pandemic started. Considering the uneven spatial distribution of those severe outcomes at the municipality level, the objective of this study was, first, to identify high-risk areas for COVID-19 hospitalisations and deaths, and second, to determine the associated contextual factors with the uneven spatial distribution of both study outcomes in Sweden.

**Methods:**

The existences of spatial autocorrelation of the standardised incidence (hospitalisations) ratio and standardised mortality ratio were investigated using Global Moran’s *I* test. Furthermore, we applied the retrospective Poisson spatial scan statistics to identify high-risk spatial clusters. The association between the contextual demographic and socioeconomic factors and the number of hospitalisations and deaths was estimated using a quasi-Poisson generalised additive regression model.

**Results:**

Ten high-risk spatial clusters of hospitalisations and six high-risk clusters of mortality were identified in Sweden from February 2020 to October 2020. The hospitalisations and deaths were associated with three contextual variables in a multivariate model: population density (inhabitants/km^2^) and the proportion of immigrants (%) showed a positive association with both outcomes, while the proportion of the population aged 65+ years (%) showed a negative association.

**Conclusions:**

Our study identified high-risk spatial clusters for hospitalisations and deaths due to COVID-19 and the association of population density, the proportion of immigrants and the proportion of people aged 65+ years with those severe outcomes. Results indicate where public health measures must be reinforced to improve sustained and future disease control and optimise the distribution of resources.

**Key questions**

**What is already known?**

- Spatial distribution of COVID-19 worldwide has shown widespread diversity in particular with regard to socioeconomic contextual factors.
- Sweden was initially one of the countries with the highest cases and death rates globally together with an alternative handling of the pandemic.
- Previous studies about COVID-19 in Sweden did not include the geospatial aspect.

**What are the new findings?**

- To the best of our knowledge, our study is the first to study the spatial distribution of severe COVID-19 and its association with contextual factors in Sweden based on a nationwide cohort consisting of 18,356 hospitalizations and 6393 deceased individuals.
- We identified ten and six high-risk spatial clusters of hospitalisations and deaths due to COVID-19, respectively.
- Hospitalisations and mortality were positively associated with population density and proportion of immigrants, while the higher the proportion of people aged 65+ years was negatively associated with both severe outcomes.

**What do the new findings imply?**

- This finding should be of high importance for policy-makers, since it indicates where the public health measures must be reinforced to improve disease control and optimise the distribution of resources to reduce hospitalisations and mortality.

## Introduction

In late 2019, an outbreak of the novel coronavirus that causes the SARS-CoV-2 occurred in China and the disease was named as COVID-19 by WHO. The virus has spread worldwide, causing more than 2 million deaths. Sweden was one of the countries with more cumulative cases and deaths per million inhabitants in Europe to autumn 2020.^1^ Certain individual characteristics have been associated with severe outcomes of the infection (hospitalisation and death), such as age, sex, ethnicity, socioeconomic status and comorbidities.^2–4^ Contextual factors such as population density^5^ or high level of urbanicity and daily commuting, as well as a greater proportion of more vulnerable minorities,^6^ and deprived areas,^4^ have been associated with high rates of cases and deaths. The presence of contextual risk factors, such as high population density in certain areas, could favour the disease spread because of the higher contact rate and physical proximity, which increase the exposure level of the population to the virus.^7^ Additionally, the risk of hospital admissions and mortality could increase in areas where the population’s vulnerability is higher because of low socioeconomic status or limited access to the healthcare system, forming spatial clusters of these events.^8^

Since contextual factors can contribute to the spatial distribution of morbidity and mortality, spatial analysis is required to identify that relationship.^6^ Previous studies have demonstrated the occurrence of spatial clustering of the disease, hospitalisations and deaths in different countries, such as Brazil,^9^ the USA,^6^ South Korea^10^ and even worldwide,^11^ identifying the high-risk regions of SARS-CoV-2 infection.

Identifying high-risk areas (clusters) and understanding the associations with contextual factors is crucial to facilitate a timely public health response, allocate and optimise resources and apply appropriate and specific intervention strategies in hotspots, among other purposes. Cluster detection is essential in epidemiological surveillance since it indicates areas with excess disease incidence, prevalence or mortality.^12^ The frequent reports of the disease in Sweden indicate that the hospitalisations and deaths do not show a homogeneous geospatial distribution in the country.^13^ However, to the best of our knowledge, no nationwide study has characterised the spatial disparity of COVID-19 hospitalisations and mortality and its association with underlying factors. Therefore, the objectives of this study were, first, to detect high-risk areas for COVID-19 severe outcomes such as hospitalisations and deaths, and second, to identify the associated contextual factors with the uneven spatial distribution of both study outcomes in Sweden.

## Methods

### Data sources

We performed an ecological study using data of confirmed hospitalisations and deaths by COVID-19 from February 2020 to 5 October 2020. COVID-19 is a notifiable disease in Sweden, and all individuals with a positive SARS-CoV-2 test (antigen or PCR positive) are reported to SmiNet (Swedish Public Health Agency) daily. The personal identification numbers from SmiNet were crosslinked with the following nationwide registers: Inpatient Register and Cause of Death Register (Swedish National Board of Health and Welfare) and LISA register (Longitudinal Integrated Database for Health Insurance and Labour Market Studies; Statistics Sweden). Through these registers, information regarding the region, municipality, date of death, age in years at diagnosis and sex was obtained. The population data from 2019 by municipalities, age and sex were obtained from Statistics Sweden (SCB).

### Determination of contextual factors

Contextual socioeconomic and demographic factors from December 2019 at municipality level including (1) population density (inhabitants/km^2^)—this variable was scaled dividing it by 100 to present the results on a more interpretable scale, (2) Gini Index, (3) mean income (thousands of SEK), (4) proportion of immigrants, (5) proportion of inhabitants older than 16 years with only compulsory education level (9 years) and (6) proportion of population aged 65+ years were obtained from Statistics Sweden (SCB). The spatial distribution of the contextual factors was displayed using choropleth maps, categorising the variables by deciles.

### Statistical analysis

#### Spatial cluster analysis

The standardised incidence (hospitalisations) ratio (SIR) and standardised mortality ratio (SMR) were calculated to show their spatial distribution in Sweden at the municipality level. SIR and SMR in this study are obtained as the ratio between observed (Oi) and expected (Ei) hospitalisations or deaths that occurred due to COVID-19. The expected hospitalisations and deaths due to COVID-19 by municipalities were adjusted for sex and age based on the total Swedish population from 2019. Eleven age groups were considered as follow: 0–4, 5–14, 15–24, 25–34, 35–44, 45–54, 55–64, 65–74, 75–84, 85–94 and ≥95. The SIR or SMR was calculated as follows:

$$SIRorSMR=\frac{Oi}{Ei}.$$

The 95% CI by municipalities was estimated using the function ‘epi.conf*’* of the package ‘epiR*’* V.2.0.17 in R V.4.0.2. The Global Moran’s I test with a simple adjacency neighbours’ matrix was used to investigate the existence of spatial autocorrelation (clustering) of the SIR and SMR. Moran’s I takes values from −1 to +1. The null hypothesis states that the variable of interest is randomly distributed across the study area, and a Moran’s I of 0 indicates complete spatial randomness. Significant indices from −1 to 0 indicate that high and low values are spatially dispersed; on the other hand, significant indices from 0 to 1 indicate that similar values are spatially clustered. To identify high-risk spatial clusters of hospitalisations and deaths, we use the retrospective Poisson spatial scan statistics with 999 Monte Carlo replications implemented in SaTScan V.9.6.^14^ This method uses moving spatial windows with different radius and a centre in each municipality centroid to scan the study area to detect spatial clusters of COVID-19 deaths. The default size of the spatial scanning windows is 50% of the population at risk, which can identify very large clusters that might not be relevant for policy-makers. Hence, the maximum spatial scanning window was set at 10% of the population at risk to avoid extremely large clusters.^15^ The null hypothesis is that deaths are randomly distributed in the space; therefore, a cluster is identified if the null hypothesis is rejected. So, if the number of observed events deaths is higher than expected, a high-risk cluster is reported.

### Contextual factor analysis

The association between the contextual factors and the number of hospitalisations and deaths was performed by using a generalised additive regression model assuming a quasi-Poisson distribution to consider overdispersion. The model used in our study was as follows: log(outcome)=predictors+offset(log(expected))+s(lon, lat)~quasi-Poisson.

The predictors are the contextual socioeconomic variables. The expected (log) number of cases standardised by sex and age of municipalities was used as an offset to estimate the standardised incidence and mortality ratios.

Spatial autocorrelation was taken into account using a Gaussian kriging smoother function—s(lon, lat)—of longitude (lon) and latitude (lat) of each municipality’s centroid^16^ following an approach similar to that of Gaudart *et al*^17^. The inclusion of the interaction term of x and y coordinates is a common way to model geospatial data together with the potential predictors. The interaction term then accounts for the spatial structure of the data.^16^ Finally, Pearson residuals of the multivariate model were tested for spatial autocorrelation using Moran’s Index.

All data cleaning, statistical analyses (except scan statistics) and cartographical displays of the results were performed using R V.4.0.2.

## Results

**Figure 1:**
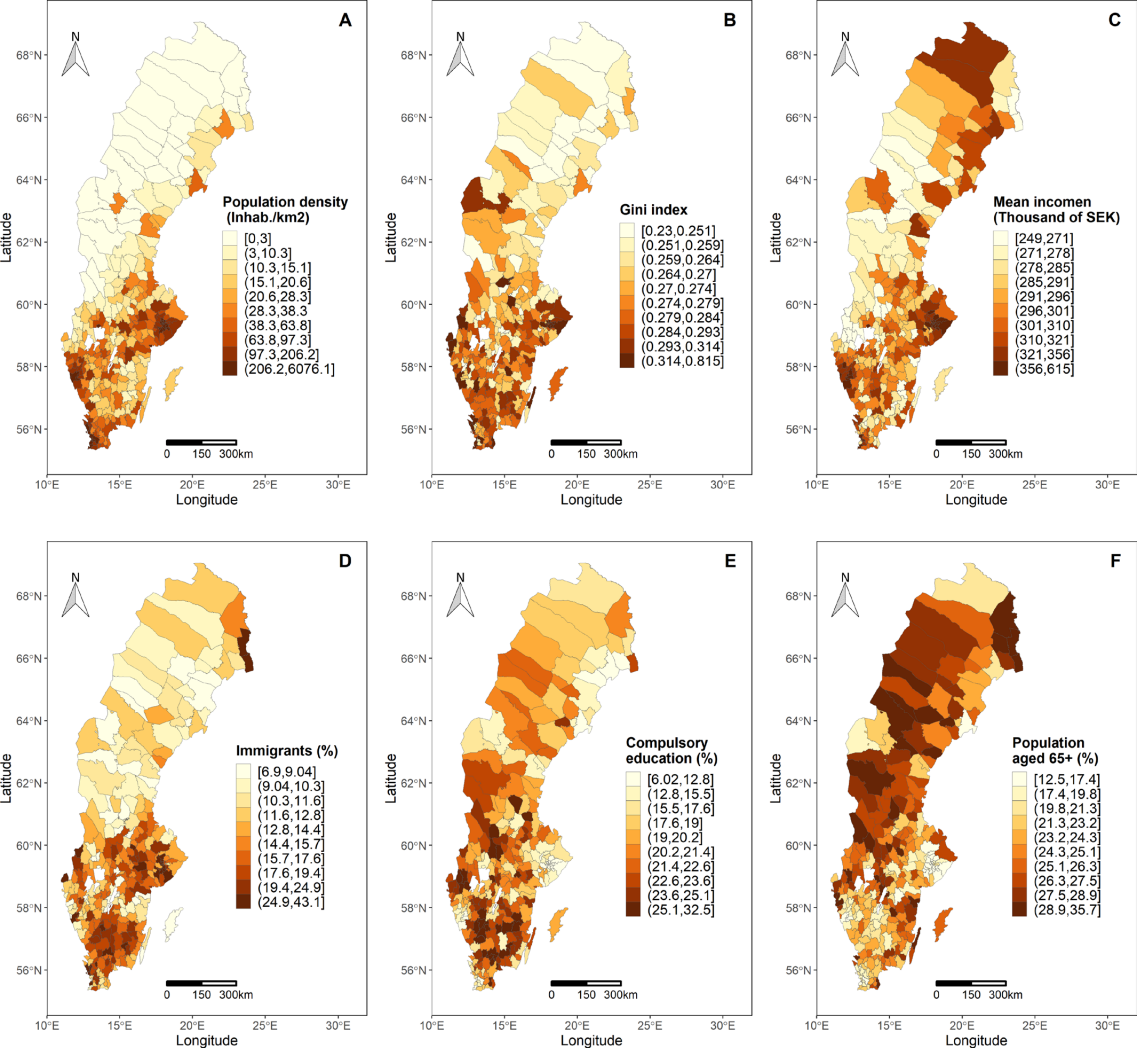
Geospatial distribution of the contextual socioeconomic and demographic variables. Population density (A), Gini Index (B), mean income (C), the proportion of immigrants (D), the proportion of inhabitants with only compulsory education (E) and the proportion of population 65+ years old (F).

Contextual socioeconomic variables are displayed on maps to show their geographical distribution (figure 1). The population density showed a median of 28.35 inhabitants per km^2^, (IQR was 12.6–81.05), the mean Gini Index was 0.28 (±0.05), the mean income was 305.93 (±41.39) thousands of SEK, the mean proportion of immigrants (%) was 15.65 (±6.43), the mean proportion of inhabitants with only compulsory education (%) was 19.67 (±4.93) and the proportion of the population aged 65+ years (%) showed a mean of 23.81 (±4.46). In general, those variables showed the highest values and their high concentration in southern municipalities except for the proportion of the population aged 65+ years. The hospitalisations and deaths due to COVID-19 were spatially aggregated by municipalities (N=290). We excluded 109 (0.59%) out of 18 356 hospitalisations and 9 (0.14%) out of 6393 deaths from the study because critical information of the individuals (sex, age or municipality) was missing.

### Spatial distribution and spatial clusters of hospitalisations and mortality

**Figure 2:**
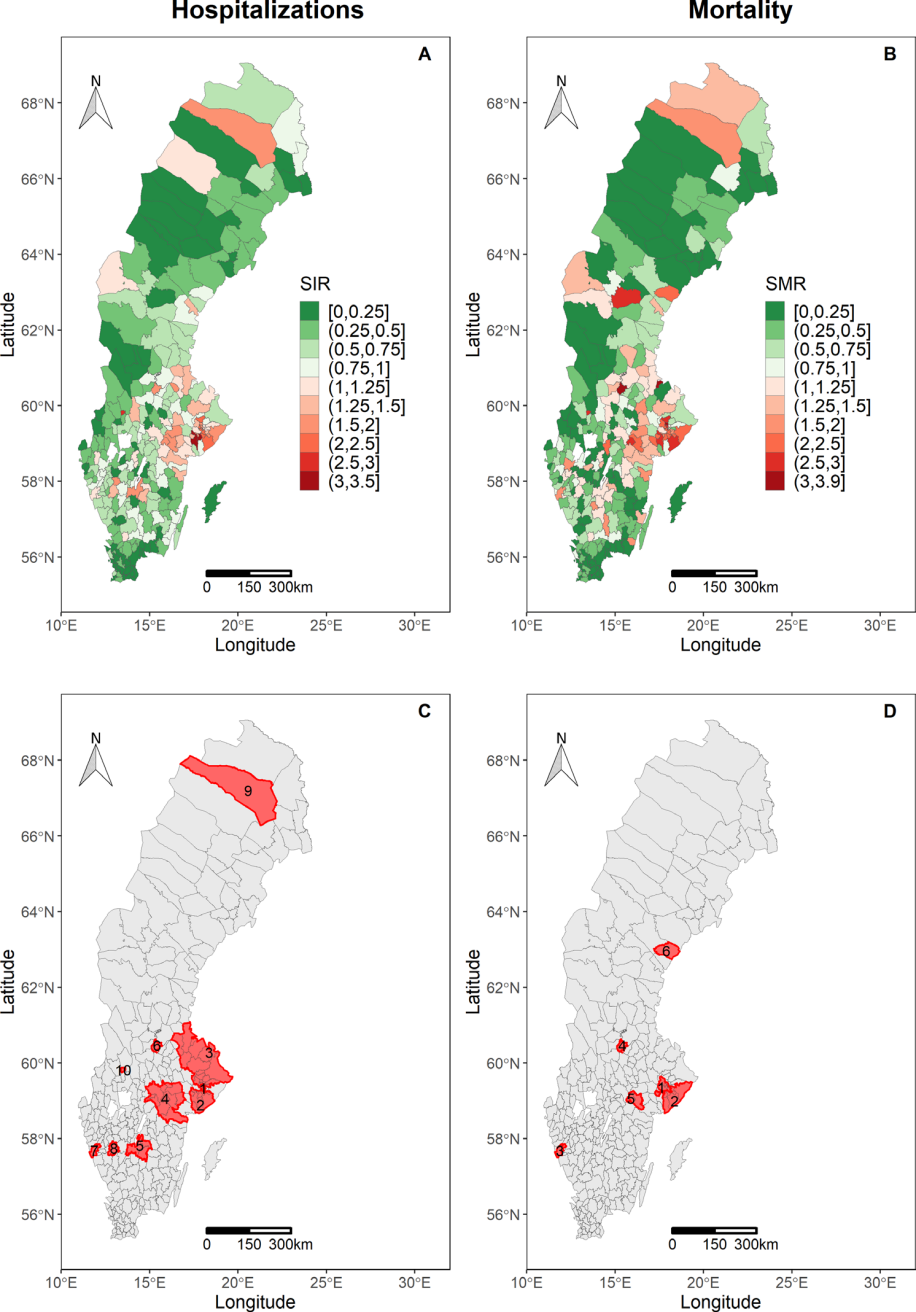
Standardisedincidence of hospitalisation ratios (SIRs) (A) and standardised mortality ratios (SMRs) (B) by municipalities and their respective spatial clusters (red areas) of COVID-19 hospitalisations (C) and deaths (D) until 5 October 2020.

The SIR and SMR by municipality showed an uneven geographical distribution over the country and in figure 2, the municipalities that have a higher or lower number of hospitalisations and deaths due to COVID-19 than expected based on the population’s demographic characteristics (age and sex) are shown. The SIR and SMR ranged from 0 to 3.5 and 0 to 3.9, respectively. SIR and SMR and their 95% CI are shown in online supplemental table A3 in online supplemental file 1, additional information such as the distribution of study contextual factors in municipalities inside and outside the spatial clusters (online supplemental table A2) and the rate ratio of hospitalisations and mortality between population younger than 65 and aged 65+ years within spatial clusters and outside spatial clusters (online supplemental table A3) is available in online supplemental file 1. The Global Moran’s I test identified the presence of spatial autocorrelation of SIR (I=0.53, p value<0.001) and SMR (I=0.43, p value<0.001), indicating the existence of spatial clustering of hospitalisations and mortality, respectively.

10.1136/bmjgh-2021-006247.supp1 Supplementary data

Ten statistically significant high-risk spatial clusters for hospitalisations were detected, most of them in southern and central Sweden. Hospitalisations cluster 1 (relative risk (RR)=2.52) includes three municipalities from Stockholm region. Cluster 2 (RR=2.63) includes eight municipalities from Stockholm region and one municipality from Södermanland region. The largest spatial cluster, cluster 3 (RR=1.34), grouped 10 municipalities from Stockholm region, 7 from Uppsala region and 2 from Gävleborgs region. Cluster 4 (RR=1.40) was the second-largest spatial cluster, including four municipalities from Södermanlands region, two from Östergötlands region, three from Örebro region and two from Västmanlands region. Three municipalities from Jönköpings region were grouped in cluster 5 (RR=1.50). Clusters 6 (RR=1.85), 7 (RR=1.25), 8 (RR=1.39), 9 (RR=1.83)—the northernmost detected cluster—and 10 (RR=2.81) include one municipality each (see figure 2C and table 1).

**Table 1:** Characteristics of the spatial clusters of COVID-19 hospitalisations and deaths

Outcome	Cluster	Number of locations	Observed (O)	Expected (E)	O/E	P value	RR (95% CI)
Hospitalisations	1	3 (Stockholm, Solna, Sundbyberg)	3808	1727.96	2.20	<0.001	2.52 (2.37 to 2.68)
	2	9 (Nynäshamn, Trosa, Tyresö, Södertälje, Nykvarn, Botkyrka, Huddinge, Salem, Haninge)	1973	805.04	2.45	<0.001	2.63 (2.41 to 2.86)
	3	19 (Östhammar, Norrtälje, Sigtuna, Upplands Väsby, Järfälla, Vaxholm, Älvkarleby, Sollentuna, Upplands-Bro, Heby, Tierp, Sandviken, Gävle, Vallentuna, Österåker, Knivsta, Uppsala, Täby, Håbo)	2158	1664.06	1.30	<0.001	1.34 (1.25 to 1.43)
	4	11 (Vingåker, Hallsberg, Örebro, Kungsör, Flen, Finspång, Katrineholm, Norrköping, Eskilstuna, Arboga, Kumla)	1315	960.20	1.37	<0.001	1.40 (1.28 to 1.52)
	5	3 (Jönköping, Aneby, Nässjö)	466	312.67	1.49	<0.001	1.50 (1.30 to 1.74)
	6	1 (Borlänge)	169	91.76	1.84	<0.001	1.85 (1.43 to 2.38)
	7	1 (Göteborg)	1114	904.31	1.23	<0.001	1.25 (1.14 to 1.37)
	8	1 (Borås)	271	196.22	1.38	0.001	1.39 (1.15 to 1.67)
	9	1 (Gällivare)	67	36.67	1.83	0.013	1.83 (1.21 to 2.71)
	10	1 (Munkfors)	24	8.56	2.80	0.022	2.81 (1.24 to 5.74)
Mortality	1	9 (Ekerö, Järfälla, Stockholm, Nykvarn, Sollentuna, Solna, Upplands-Bro, Sundbyberg, Salem)	1565	637.99	2.45	<0.001	2.92 (2.65 to 3.23)
	2	8 (Haninge, Nynäshamn, Lidingö, Tyresö, Nacka, Värmdö, Botkyrka, Huddinge)	572	266.36	2.15	<0.001	2.26 (1.95 to 2.69)
	3	1 (Göteborg)	451	277.97	1.62	<0.001	1.67 (1.43 to 1.95)
	4	1 (Borlänge)	98	32.59	3.01	<0.001	3.04 (2.02 to 4.45)
	5	2 (Vingåker, Katrineholm)	73	32.05	2.28	<0.001	2.29 (1.51 to 3.48)
	6	1 (Kramfors)	36	16.15	2.23	0.021	2.24 (1.25 to 4.07)

RR, relative risk.

In addition, six statistically significant high-risk mortality clusters were identified, all of them in southern and central Sweden. Cluster 1 (RR 2.92) and cluster 2 (RR=2.26) were formed by nine and eight municipalities, respectively, from Stockholm region. Cluster 3 (RR=1.67), 4 (RR=3.04) and 6 (RR=2.24) included one single municipality each and cluster 5 (RR=2.29) comprised two municipalities from Södermanlands region (see figure 2D and table 1).

### Association of hospitalisations and mortality with contextual factors

In the univariate analyses (table 2), all contextual factors, except the Gini Index, showed an association with the ratio of hospitalisations by municipalities in Sweden. However, in the multivariate model, the population density (RR=1.005, 95% CI 1.002 to 1.009) and the proportion of immigrants (RR=1.026, 95% CI 1.016 to 1.037) showed a positive association with hospitalisations, while the proportion of population 65 years and older (RR=0.949, 95% CI 0.929 to 0.969) showed a significantly negative influence on hospitalisations in Sweden. This multivariate model explained 90.4% of the COVID-19 hospitalisation, while the residuals did not show spatial autocorrelation (Moran’s I=−0.037, p value=0.411).

**Table 2:** Univariate and multivariate models for COVID-19 hospitalisations and deaths rate at the municipality level in Sweden

Outcome	Variables	Univariate models		Multivariate model	
		RR (95% CI)	P value	RR (95% CI)	P value
Hospitalisations	Population density (Inhab./km^2^)	**1.008 (1.004 to 1.012)**	**<0.001**	**1.005 (1.002 to 1.009)**	**0.004**
	Gini Index	0.707 (0.243 to 2.059)	0.5260	1.687 (0.571 to 4.987)	0.345
	Mean income (thousands of SEK)	**0.996 (0.995 to 0.998)**	**<0.001**	1.001 (0.999 to 1.002)	0.603
	Proportion of immigrants (%)	**1.041 (1.034 to 1.048)**	**<0.001**	**1.026 (1.016 to 1.037)**	**<0.001**
	Proportion of inhabitants with compulsory education (%)	**1.016 (1.000 to 1.031)**	**0.0495**	1.019 (1.000 to 1.039)	0.057
	Proportion of population 65+ years (%)	**0.925 (0.911 to 0.939)**	**<0.001**	**0.949 (0.929 to 0.969)**	**<0.001**
Mortality	Population density (Inhab./km^2^)	**1.008 (1.002 to 1.014)**	**0.0063**	**1.006 (1.000 to 1.012)**	**0.039**
	Gini Index	0.472 (0.106 to 2.110)	0.327	0.906 (0.140 to 5.843)	0.917
	Mean income (thousands of SEK)	**0.996 (0.994 to 0.998)**	**<0.001**	1.001 (0.998 to 1.004)	0.454
	Proportion of immigrants (%)	**1.044 (1.033 to 1.056)**	**<0.001**	**1.031 (1.013 to 1.048)**	**0.001**
	Proportion of inhabitants with compulsory education (%)	1.021 (0.999 to 1.043)	0.0665	1.026 (0.994 to 1.058)	0.114
	Proportion of population 65+ years (%)	**0.931** **(0.911 to 0.952)**	**<0.001**	**0.956 (0.924 to 0.989**)	**0.010**

Values in bold represent statistically significant relative risk (RR).

The univariate analyses (table 2) with mortality as a dependent variable similarly showed only the Gini Index and education were associated with this outcome. In the multivariate model, population density (RR=1.006, 95% CI 1.000 to 1.012) and the proportion of immigrants (RR=1.031, 95% CI 1.013 to 1.048) remained positively associated with the mortality, while the proportion of population 65 years and older (RR=0.956, 95% CI 0.924 to 0.989) was significantly negatively associated with mortality explaining the 76.5% of the deviance in the model. Spatial autocorrelation of the residuals was not found (Moran’s I=−0.045, p value=0.302).

## Discussion

Our study demonstrated that the COVID-19 hospitalisations and deaths in Sweden were spatially clustered, first by the Global Moran’s I and later by using the scan statistic method that allows identifying the clusters’ location. The higher COVID-19 hospitalisations and mortality standardised ratios were more frequent in southern Sweden, but the municipality of Gällivare in the Norrbotten region, in northernmost Sweden, also showed high hospitalisation and mortality ratios. The spatial clusters (high rate) indicate the presence of high-risk areas, where the number of hospitalisations and deaths is significantly higher than expected.

The spatial clusters identified in our study could be related to a high viral transmission rate at the local level (eg, municipalities, regions), leading to the rise of the disease incidence, causing higher hospitalisation and mortality rates. Our findings are supported by previous studies at the national, European and worldwide level that found geographic clusters of COVID-19 infections, hospitalisations and deaths.^11 18 19^ The clustering of risk factors or a combination of risk factors, such as high population density and a high proportion of the vulnerable population, can influence the spatial clustering of COVID-19, which creates an increased risk in its immediate neighbouring municipalities. Population density is a factor commonly associated with infectious disease transmission,^20^ and since COVID-19 is mainly transmitted from person to person, disease transmission likely favours densely populated areas where face-to-face interaction among residents frequently occurs. This would create hotspots due to the rapid spread. This is exemplified by Kim and Castro^10^ in South Korea who reported that COVID-19 clusters were detected in densely populated districts, therefore it is also a likely explanation for our findings in this study.

Another explanation for our findings could be the mobility of residents in highly densely populated areas, which could facilitate the introduction, spread and persistence of COVID-19. Studies performed in Sweden have shown how the risk of deaths increases for individuals living in highly densely populated areas.^5^ Population density could therefore be a proxy for a high contact rate because of mobility rather than physical proximity.^21^ The Northern municipality of Gällivare in the Norrbotten region illustrates this case since it is a municipality with a low population density but showed high hospitalisation and mortality ratios. Gällivare is located in a major iron ore mining area, where a considerable circulation of people coming from different regions could facilitate the spread of the disease, resulting in an increase of hospitalisations and deaths and the spatial clustering in that municipality.

Our study found that areas with a higher proportion of immigrants were at increased risk of hospitalisation or death due to COVID-19. This finding is corroborated by other individual-level studies that have shown that immigrants, in general, have a higher risk of being hospitalised^4^ or dying^4 5 22^ by COVID-19 compared with the natives and places with high shares of immigrants are more susceptible to the virus spread, increasing severe outcomes.^5^ Potential explanatory factors for this finding could be overcrowding and multigenerational households, in addition to other risk factors such as low incomes, work in front-line activities that increase the exposure to the virus and language limitations might have undermined the possibility to adopt preventive measures.^5^ Social vulnerability has been strongly associated with the presence of spatial clusters of confirmed cases and mortality in other settings.^23^ In addition, socially disadvantaged individuals might have less timely access to medical services,^24^ which likely increases the probability of severe or fatal COVID-19.^25^ Though the Swedish healthcare system offers theoretically equal access to the population with residence permit, there are still significant barriers to access among the migrant population, such as the lack of understanding of the national language, financial difficulties, cultural differences and lack of knowledge about the functioning of the healthcare system.^26 27^

On the other hand, the Swedish healthcare system has been moving, particularly in the last two decades, into a market-orientation model, which can create a disadvantage in access for the poorest fraction of the population, including migrants.^28 29^ This could have exacerbated during the pandemic. Additionally, other potential factors such as the perception among migrants about the quality of care, trust on the health services and the responsiveness of the health system could influence care-seeking behaviour and thus explain some of the observed findings.^30^

Interestingly, the highest numbers of hospitalisations and deaths by COVID-19 occurred in areas with a lower proportion of the population aged 65+ years. It does not mean that people younger than 65 years have a higher risk of having a severe outcome due to COVID-19; in fact, the rate ratio of hospitalisation and deaths among people aged 65+ years were significantly higher than among the population younger than 65 years even outside or inside the spatial clusters (online supplemental table A3). Multiple studies have demonstrated that age is a risk factor for hospitalisation and deaths by COVID-19, particularly in people older than 65 years.^2 3 31^ However, at the spatial (municipality) level, other variables can be more important in determining the spread of the virus and the distribution of those outcomes, such as population density, mobility, higher socioeconomic activities and social contact, and that tend to be lower in areas with a higher proportion of older people.^32^

We found in the univariate models a protective effect of mean income per municipality to hospitalisation and mortality ratios, but it was not significant when adjusted for other variables. However, in Sweden, studies performed at the individual level and without a spatial approach have found an association between income and deaths by COVID-19 adjusting for multiple socioeconomic variables and other individual characteristics (age and sex), showing that the more disadvantaged people have a higher risk of dying.^5 22^ So, although the mean income at the municipality level was not significant, this finding must be interpreted with caution because the economic level could play a certain role in COVID-19-related hospitalisations and deaths. In this line, the proportion of inhabitants with compulsory education showed a significant positive effect in the univariable hospitalisations and mortality spatial regression models, but although the variable showed a similar effect in the multivariable models, it was not significant. Other studies have found an association between lower education level and hospitalisations and mortality,^5 22 33^ we must not ignore the effect of this contextual variable that is also related to income and level of exposure to COVID-19 due to occupation characteristics (eg, blue collar).^33^

To the best of our knowledge, this is the first nationwide study in Sweden that identifies geographical hospitalisations and mortality clusters and associated contextual factors with COVID-19 severity outcomes. The current study was based on nationwide data with a high level of completeness obtained from reliable registers, which allows for precise and reliable results.

Still, we must acknowledge some limitations. Connectivity or mobility was not considered, which could be an important factor that helps to explain more specifically the occurrence of hospitalisations and deaths as a result of high disease transmission. Other relevant factors such as occupation (eg, proportion of blue-collar and white-collar occupations), adherence to control measures (eg, social distancing, wearing face masks, risk communication and community engagement), comorbidities and its spatial distribution among municipalities could not be analysed because this information was not available. In this line, data on healthcare utilisation, quality of care and perception of the healthcare by population groups and municipalities were not available, so its potential role could not be considered. Though COVID-19 hospitalised individuals and deaths are less influenced by testing rates, there exists the possibility that certain COVID-19 deaths were not detected leading to a different pattern among municipalities. Furthermore, the scan statistics method implanted in SaTScan software has certain intrinsic limitations. The results are sensitive to the parameter settings in running SaTScan; thus, modifying the size of the study area or the maximum size of the spatial windows can change the results.^34^ Additionally, the scan statistic does not capture irregular-shaped clusters well due to its circular scanning window^35 36^ and it could decrease statistical power.^37^ Finally, being an ecological study, caution should be taken when making inferences at the individual level and interpreting results as a causal effect.

In conclusion, our study identified high-risk clusters for hospitalisations and deaths due to COVID-19 and the positive association of those severe outcomes with population density and the proportion of immigrants in Sweden, while the proportion of the population aged 65+ years was negatively associated with both outcomes. Thus, this study complements the knowledge about COVID-19 hospitalisations and mortality obtained from research at the individual level by showing the effect of contextual factors from an ecological perspective. This finding should be of high importance for policy-makers, since it indicates where the public health measures must be reinforced to improve disease control and optimise the distribution of resources to reduce hospitalisations and mortality.

## Data Availability

No data are available. The study used secondary registry data which are regulated by the Public Access to Information and Secrecy Act (2009:400) and are protected by strict confidentiality. For the purpose of research though, after formal application to access personal data, the responsible authority can grant access to data, though this is contingent on vetting by the Ethical Review Authority of Sweden, according to the Act (2003:460) concerning the Ethical Review of Research Involving Humans.
