# Supplementary material for: Spatial clustering and contextual factors associated with hospitalisation and deaths due to COVID-19 in Sweden: a geospatial nationwide ecological study
Source: BMJ Glob Health. 2021 Jul 28;6(7):e006247. doi: 10.1136/bmjgh-2021-006247 (PMC8322019; doi:10.1136/bmjgh-2021-006247)
Supplement: Supplementary data [file bmjgh-2021-006247supp001.pdf]

Spatial clustering and contextual factors associated with hospitalization and deaths due to COVID-19 in Sweden: a geospatial nationwide ecological study

Osvaldo Fonseca-Rodríguez[1,*], Per E. Gustafsson[2], Miguel San Sebastian[2], Anne-Marie Fors Connolly[1]

[1]Department of Clinical Microbiology, Umeå University, 901 85 Umeå, Sweden
[2]Department of Epidemiology and Global Health, Umeå University, 901 87 Umeå, Sweden

*Corresponding author:
Osvaldo Fonseca-Rodríguez
Email: osvaldo.fonseca@umu.se

Table A1. Standardized Incidence of Hospitalizations (SIR) and Standardized Mortality Ratios (SMR) by municipalities in Sweden (February 2020 to October 5th 2020)

Municipality	SIR (95%CI)	SMR (95%CI)
**Norrtälje**	**0.59 (0.46-0.73)**	**0.62 (0.42-0.88)**
**Sigtuna**	**2.3 (1.97-2.68)**	**2.77 (2.1-3.58)**
**Nynäshamn**	0.93 (0.69-1.23)	0.84 (0.47-1.38)
**Varberg**	**0.62 (0.49-0.77)**	**0.53 (0.34-0.78)**
**Krokom**	**0.4 (0.2-0.72)**	**0 (0-0.38)**
**Upplands Väsby**	**1.8 (1.51-2.13)**	**3.38 (2.65-4.24)**
**Trosa**	0.62 (0.35-1.01)	0.46 (0.13-1.19)
**Gnosjö**	0.57 (0.27-1.04)	1.87 (0.97-3.27)
**Gotland**	**0.16 (0.1-0.25)**	**0.11 (0.04-0.26)**
**Hässleholm**	**0.43 (0.31-0.57)**	**0.48 (0.3-0.75)**
**Tibro**	1.08 (0.69-1.6)	1.36 (0.7-2.37)
**Ulricehamn**	0.91 (0.66-1.22)	**0.47 (0.22-0.9)**
**Mariestad**	**0.26 (0.14-0.44)**	**0.1 (0.01-0.36)**
**Hofors**	**1.56 (1.06-2.21)**	0.66 (0.21-1.53)
**Hudiksvall**	**0.75 (0.57-0.98)**	0.67 (0.4-1.04)
**Sollefteå**	**0.48 (0.29-0.74)**	0.75 (0.4-1.28)
**Lycksele**	**0.08 (0.01-0.3)**	**0 (0-0.39)**
**Järfälla**	**2.32 (2.06-2.6)**	**2.32 (1.88-2.83)**
**Nyköping**	1.1 (0.91-1.31)	1.28 (0.96-1.66)
**Växjö**	**0.48 (0.38-0.61)**	**0.56 (0.38-0.79)**
**Högsby**	**0.33 (0.09-0.84)**	**0 (0-0.76)**
**Åstorp**	**0.38 (0.18-0.7)**	0.35 (0.07-1.03)
**Sotenäs**	**0.23 (0.08-0.55)**	**0.23 (0.03-0.82)**
**Mellerud**	**0.4 (0.17-0.79)**	0.73 (0.27-1.58)
**Hallsberg**	**0.54 (0.31-0.87)**	0.47 (0.15-1.09)
**Ånge**	**0.54 (0.27-0.97)**	0.37 (0.08-1.07)
**Kramfors**	0.87 (0.61-1.21)	**2.23 (1.56-3.09)**
**Överkalix**	**0 (0-0.45)**	0 (0-1.07)
**Luleå**	**0.4 (0.3-0.52)**	**0.12 (0.05-0.27)**
**Lidingö**	1.17 (0.96-1.42)	**1.79 (1.37-2.29)**

**Sävsjö**	**0.49 (0.24-0.87)**	**0.34 (0.07-0.98)**
**Herrljunga**	**1.56 (1.04-2.24)**	1.14 (0.49-2.25)
**Kungälv**	**0.78 (0.6-0.99)**	**0.57 (0.33-0.91)**
**Örebro**	**1.24 (1.11-1.39)**	1.05 (0.84-1.29)
**Jokkmokk**	**0 (0-0.34)**	**0 (0-0.89)**
**Övertorneå**	0.84 (0.39-1.6)	0.65 (0.13-1.89)
**Vaxholm**	1.18 (0.76-1.74)	0.44 (0.09-1.3)
**Ödeshög**	**0.09 (0-0.51)**	0.24 (0.01-1.32)
**Åtvidaberg**	**0.46 (0.23-0.83)**	0.65 (0.24-1.41)
**Gislaved**	0.74 (0.53-1.01)	**0.51 (0.24-0.93)**
**Partille**	0.8 (0.6-1.05)	0.73 (0.42-1.18)
**Bräcke**	**0.22 (0.04-0.63)**	**2.85 (1.59-4.69)**
**Malå**	0.46 (0.1-1.35)	0.39 (0.01-2.19)
**Pajala**	0.9 (0.48-1.55)	0.67 (0.18-1.71)
**Habo**	0.87 (0.51-1.4)	**0.17 (0-0.94)**
**Vaggeryd**	**0.08 (0.01-0.29)**	1.14 (0.55-2.09)
**Tjörn**	**0.6 (0.37-0.93)**	0.48 (0.18-1.05)
**Vansbro**	**0.28 (0.08-0.71)**	0.69 (0.19-1.76)
**Ovanåker**	0.61 (0.34-1.01)	1.26 (0.65-2.2)
**Stockholm**	**2.22 (2.15-2.3)**	**2.46 (2.32-2.61)**
**Olofström**	**0.18 (0.06-0.41)**	**0.17 (0.02-0.62)**
**Bjuv**	**0.08 (0.01-0.27)**	**0 (0-0.44)**
**Höör**	**0.1 (0.02-0.29)**	**0.09 (0-0.52)**
**Tomelilla**	**0.11 (0.02-0.32)**	**0.1 (0-0.53)**
**Falkenberg**	**0.57 (0.43-0.75)**	0.69 (0.44-1.02)
**Essunga**	0.52 (0.19-1.14)	1.13 (0.37-2.63)
**Eda**	**0.23 (0.06-0.59)**	**0 (0-0.54)**
**Nordmaling**	**0.13 (0.02-0.48)**	**0 (0-0.63)**
**Vindeln**	0.43 (0.14-1.01)	0.62 (0.13-1.81)
**Piteå**	**0.32 (0.21-0.48)**	**0.39 (0.2-0.7)**
**Tyresö**	**1.87 (1.58-2.2)**	**2.28 (1.73-2.96)**
**Södertälje**	**3.5 (3.21-3.8)**	**2.7 (2.26-3.2)**
**Östhammar**	1.03 (0.76-1.36)	1.16 (0.73-1.76)
**Gnesta**	1.23 (0.81-1.81)	**2.08 (1.16-3.42)**
**Hultsfred**	**0.33 (0.16-0.61)**	**0.16 (0.02-0.58)**
**Borås**	**1.38 (1.22-1.56)**	1.17 (0.93-1.45)
**Borlänge**	**1.84 (1.57-2.14)**	**3.01 (2.44-3.66)**
**Storuman**	**0.15 (0.02-0.55)**	**0 (0-0.68)**
**Boden**	0.74 (0.53-1)	0.77 (0.44-1.24)
**Nykvarn**	1.36 (0.87-2.02)	**2.22 (1.11-3.98)**
**Älvkarleby**	0.86 (0.49-1.39)	**3.01 (1.86-4.6)**
**Vingåker**	**1.72 (1.17-2.44)**	**2.64 (1.54-4.23)**
**Karlshamn**	**0.15 (0.07-0.28)**	**0.16 (0.04-0.4)**
**Orust**	**0.3 (0.14-0.54)**	**0.3 (0.08-0.77)**

**Grästorp**	0.51 (0.19-1.11)	0.22 (0.01-1.21)
**Falköping**	**0.51 (0.35-0.72)**	**0.25 (0.09-0.54)**
**Lindesberg**	**0.5 (0.31-0.74)**	0.57 (0.27-1.05)
**Sala**	0.85 (0.6-1.16)	**1.7 (1.14-2.44)**
**Östersund**	**0.69 (0.55-0.86)**	0.85 (0.59-1.18)
**Bjurholm**	0.35 (0.04-1.28)	0 (0-1.29)
**Robertsfors**	**0.36 (0.12-0.84)**	0.56 (0.12-1.64)
**Uppvidinge**	0.83 (0.48-1.35)	0.38 (0.08-1.1)
**Mönsterås**	0.84 (0.53-1.26)	1.42 (0.79-2.34)
**Burlöv**	0.71 (0.44-1.07)	1.12 (0.56-2.01)
**Örkelljunga**	**0.29 (0.11-0.64)**	0.38 (0.08-1.11)
**Alingsås**	**0.69 (0.52-0.9)**	**0.45 (0.24-0.78)**
**Torsby**	**0.38 (0.18-0.7)**	**0.27 (0.06-0.79)**
**Munkfors**	**2.8 (1.8-4.17)**	**2.65 (1.27-4.87)**
**Surahammar**	0.6 (0.31-1.05)	**0.13 (0-0.75)**
**Sundsvall**	0.85 (0.72-1)	**0.75 (0.56-0.99)**
**Åre**	1.03 (0.63-1.59)	1.36 (0.59-2.68)
**Oxelösund**	**1.47 (1.03-2.03)**	1.07 (0.54-1.92)
**Jönköping**	**1.52 (1.36-1.68)**	1.01 (0.81-1.24)
**Lessebo**	**0.37 (0.13-0.8)**	0.49 (0.1-1.42)
**Mörbylånga**	**0.56 (0.32-0.89)**	0.45 (0.15-1.06)
**Nybro**	**0.53 (0.33-0.8)**	**0.3 (0.1-0.7)**
**Perstorp**	**0.29 (0.08-0.74)**	0.41 (0.05-1.47)
**Götene**	**0.19 (0.06-0.44)**	**0.1 (0-0.58)**
**Kungsör**	0.78 (0.42-1.33)	**0.17 (0-0.95)**
**Malung-Sälen**	**0.19 (0.05-0.48)**	**0.25 (0.03-0.9)**
**Kiruna**	**0.61 (0.4-0.89)**	1.5 (0.94-2.27)
**Nacka**	**1.49 (1.31-1.69)**	**1.99 (1.62-2.42)**
**Flen**	**1.4 (1.03-1.85)**	1.33 (0.79-2.11)
**Mjölby**	**0.53 (0.35-0.77)**	0.98 (0.58-1.55)
**Borgholm**	**0.41 (0.21-0.74)**	0.44 (0.14-1.02)
**Simrishamn**	**0.13 (0.05-0.29)**	**0.1 (0.01-0.37)**
**Laholm**	**0.37 (0.22-0.58)**	0.61 (0.32-1.07)
**Dals-Ed**	0.4 (0.11-1.03)	0.77 (0.16-2.25)
**Gullspång**	**0.25 (0.05-0.72)**	0.2 (0.01-1.13)
**Vänersborg**	0.78 (0.59-1)	**1.58 (1.15-2.12)**
**Fagersta**	0.65 (0.38-1.04)	0.84 (0.38-1.59)
**Alvesta**	0.87 (0.6-1.23)	**1.7 (1.1-2.51)**
**Älmhult**	0.88 (0.58-1.27)	0.98 (0.51-1.71)
**Karlskrona**	**0.44 (0.33-0.57)**	**0.2 (0.09-0.37)**
**Ängelholm**	**0.21 (0.13-0.34)**	**0.27 (0.12-0.51)**
**Kungsbacka**	**0.61 (0.49-0.75)**	**0.39 (0.24-0.6)**
**Skövde**	0.89 (0.71-1.1)	0.93 (0.63-1.31)
**Storfors**	0.69 (0.26-1.51)	0.31 (0.01-1.72)

**Mora**	**0.14 (0.05-0.31)**	**0.06 (0-0.34)**
**Danderyd**	1.04 (0.8-1.33)	0.73 (0.43-1.15)
**Finspång**	1.01 (0.73-1.35)	1.42 (0.91-2.11)
**Sollentuna**	**1.58 (1.36-1.82)**	**2.77 (2.26-3.36)**
**Katrineholm**	**1.79 (1.48-2.15)**	**2.19 (1.65-2.84)**
**Vadstena**	**0.23 (0.06-0.6)**	0.7 (0.23-1.62)
**Kalmar**	0.92 (0.76-1.11)	**0.3 (0.16-0.51)**
**Svalöv**	**0.21 (0.07-0.48)**	**0.26 (0.03-0.95)**
**Svedala**	**0.28 (0.13-0.51)**	**0.35 (0.1-0.9)**
**Sjöbo**	**0.16 (0.06-0.35)**	**0 (0-0.28)**
**Vara**	**0.19 (0.07-0.41)**	**0 (0-0.32)**
**Töreboda**	1.1 (0.68-1.68)	1.21 (0.56-2.31)
**Mölndal**	1.01 (0.83-1.21)	1.01 (0.7-1.41)
**Arvika**	**0.41 (0.26-0.62)**	**0.13 (0.03-0.39)**
**Sorsele**	**0 (0-0.64)**	0 (0-1.48)
**Solna**	**1.83 (1.6-2.08)**	**2.13 (1.7-2.63)**
**Strömstad**	**0.16 (0.04-0.41)**	**0.21 (0.03-0.77)**
**Tidaholm**	**0.47 (0.24-0.82)**	0.5 (0.16-1.16)
**Forshaga**	**0.41 (0.19-0.78)**	0.62 (0.2-1.44)
**Ludvika**	0.87 (0.63-1.16)	1.14 (0.73-1.69)
**Örnsköldsvik**	**0.34 (0.24-0.46)**	**0.22 (0.1-0.41)**
**Gällivare**	**1.83 (1.42-2.32)**	**1.9 (1.25-2.76)**
**Upplands-Bro**	**1.48 (1.14-1.88)**	**2.45 (1.64-3.52)**
**Tranås**	**0.4 (0.22-0.66)**	**0.13 (0.02-0.46)**
**Tingsryd**	0.94 (0.61-1.37)	0.96 (0.5-1.68)
**Sölvesborg**	**0.17 (0.06-0.36)**	**0.07 (0-0.4)**
**Kävlinge**	**0.13 (0.05-0.27)**	**0.12 (0.01-0.43)**
**Hörby**	**0.17 (0.05-0.39)**	**0.09 (0-0.51)**
**Munkedal**	**0.38 (0.16-0.74)**	0.59 (0.19-1.38)
**Färgelanda**	1.12 (0.63-1.85)	0.4 (0.05-1.43)
**Bollebygd**	**0.3 (0.1-0.69)**	**0 (0-0.66)**
**Uddevalla**	**0.69 (0.54-0.87)**	0.78 (0.52-1.11)
**Skinnskatteberg**	**0.1 (0-0.58)**	0 (0-1.01)
**Norsjö**	**0.12 (0-0.66)**	0 (0-1.15)
**Värmdö**	**1.65 (1.37-1.97)**	**2.32 (1.7-3.08)**
**Sundbyberg**	**2.49 (2.15-2.88)**	**3.9 (3.08-4.87)**
**Heby**	**0.36 (0.17-0.66)**	0.47 (0.15-1.1)
**Boxholm**	0.62 (0.25-1.27)	**0 (0-0.86)**
**Norrköping**	**1.45 (1.31-1.61)**	**1.29 (1.05-1.55)**
**Aneby**	1.24 (0.71-2.01)	1.47 (0.59-3.03)
**Härryda**	**0.62 (0.44-0.85)**	0.69 (0.37-1.19)
**Trollhättan**	0.95 (0.77-1.16)	1.08 (0.77-1.47)
**Årjäng**	**0.1 (0.01-0.36)**	**0 (0-0.48)**
**Hagfors**	**0.37 (0.18-0.68)**	**0.18 (0.02-0.65)**

**Leksand**	0.83 (0.55-1.2)	1.09 (0.61-1.8)
**Ockelbo**	1.38 (0.8-2.21)	1.32 (0.49-2.88)
**Härnösand**	**0.72 (0.5-0.99)**	0.72 (0.4-1.22)
**Haparanda**	**0.2 (0.05-0.5)**	**0 (0-0.5)**
**Tierp**	**0.53 (0.33-0.8)**	**0.25 (0.07-0.63)**
**Enköping**	0.94 (0.74-1.18)	0.87 (0.56-1.28)
**Ydre**	0.49 (0.13-1.24)	0 (0-1.14)
**Valdemarsvik**	1.37 (0.87-2.03)	1.53 (0.77-2.74)
**Ljungby**	**0.55 (0.37-0.78)**	0.68 (0.38-1.12)
**Bromölla**	**0.16 (0.04-0.41)**	**0.11 (0-0.61)**
**Malmö**	**0.65 (0.58-0.72)**	**0.48 (0.38-0.6)**
**Eslöv**	**0.34 (0.21-0.53)**	**0.25 (0.08-0.59)**
**Halmstad**	**0.39 (0.31-0.49)**	**0.22 (0.12-0.37)**
**Göteborg**	**1.23 (1.16-1.31)**	**1.62 (1.48-1.78)**
**Askersund**	0.66 (0.38-1.07)	0.65 (0.24-1.42)
**Sandviken**	1.02 (0.81-1.28)	1.17 (0.8-1.64)
**Arvidsjaur**	**0.45 (0.17-0.99)**	0.38 (0.05-1.39)
**Arjeplog**	1.11 (0.45-2.28)	0 (0-1.39)
**Kalix**	**0.17 (0.06-0.37)**	**0.15 (0.02-0.52)**
**Vårgårda**	0.62 (0.33-1.05)	0.82 (0.3-1.78)
**Sunne**	**0.36 (0.17-0.67)**	0.66 (0.26-1.35)
**Karlstad**	**0.56 (0.45-0.68)**	**0.3 (0.18-0.47)**
**Lekeberg**	0.54 (0.24-1.07)	**0 (0-0.7)**
**Laxå**	0.64 (0.27-1.25)	**2.3 (1.19-4.02)**
**Nora**	**0.23 (0.08-0.54)**	**0 (0-0.46)**
**Gävle**	**1.27 (1.11-1.45)**	1.18 (0.93-1.49)
**Strömsund**	**0.15 (0.04-0.4)**	**0.28 (0.06-0.82)**
**Vallentuna**	0.85 (0.62-1.13)	0.69 (0.34-1.23)
**Österåker**	1.1 (0.88-1.36)	0.96 (0.61-1.44)
**Botkyrka**	**3.22 (2.93-3.54)**	**2.37 (1.89-2.93)**
**Värnamo**	0.88 (0.67-1.14)	1.09 (0.71-1.6)
**Oskarshamn**	**0.43 (0.27-0.65)**	**0.25 (0.08-0.58)**
**Helsingborg**	**0.6 (0.51-0.71)**	0.9 (0.72-1.13)
**Tranemo**	0.8 (0.48-1.25)	0.64 (0.24-1.4)
**Lidköping**	**0.46 (0.32-0.63)**	0.88 (0.57-1.3)
**Kil**	**0.17 (0.05-0.44)**	**0.11 (0-0.63)**
**Berg**	0.65 (0.31-1.19)	1.11 (0.45-2.28)
**Emmaboda**	**0.2 (0.05-0.5)**	**0 (0-0.43)**
**Lund**	**0.25 (0.18-0.33)**	**0.31 (0.19-0.48)**
**Lilla Edet**	0.8 (0.49-1.24)	1.12 (0.51-2.12)
**Åmål**	**0.49 (0.26-0.84)**	**0.36 (0.1-0.92)**
**Hällefors**	0.59 (0.27-1.12)	1.08 (0.43-2.23)
**Hedemora**	0.67 (0.41-1.02)	**0.42 (0.14-0.98)**
**Bollnäs**	**0.71 (0.5-0.97)**	**0.44 (0.2-0.83)**

**Skellefteå**	**0.33 (0.25-0.44)**	**0.29 (0.16-0.48)**
**Ekerö**	1.12 (0.84-1.46)	1.04 (0.58-1.72)
**Knivsta**	0.97 (0.64-1.43)	0.72 (0.23-1.67)
**Vetlanda**	**0.68 (0.48-0.93)**	**0.45 (0.22-0.83)**
**Huddinge**	**2.34 (2.12-2.59)**	**2.43 (1.99-2.93)**
**Uppsala**	**1.32 (1.2-1.44)**	**1.41 (1.2-1.64)**
**Linköping**	0.94 (0.82-1.06)	1.02 (0.82-1.24)
**Markaryd**	1.06 (0.66-1.6)	1.17 (0.56-2.16)
**Ronneby**	**0.2 (0.11-0.36)**	**0.17 (0.05-0.44)**
**Staffanstorp**	**0.21 (0.1-0.4)**	**0.14 (0.02-0.5)**
**Östra Göinge**	**0.32 (0.15-0.61)**	**0.28 (0.06-0.83)**
**Trelleborg**	**0.33 (0.22-0.48)**	**0.3 (0.14-0.57)**
**Bengtsfors**	**0.42 (0.19-0.79)**	**0.33 (0.07-0.96)**
**Grums**	0.65 (0.33-1.13)	0.7 (0.23-1.64)
**Härjedalen**	**0.36 (0.15-0.7)**	**0.11 (0-0.6)**
**Vilhelmina**	**0 (0-0.27)**	**0 (0-0.7)**
**Salem**	**1.75 (1.29-2.32)**	**1.97 (1.17-3.11)**
**Skurup**	**0.25 (0.1-0.51)**	**0.1 (0-0.58)**
**Ystad**	**0.13 (0.05-0.25)**	**0.04 (0-0.22)**
**Kristianstad**	**0.25 (0.18-0.34)**	**0.24 (0.13-0.4)**
**Hylte**	**0.1 (0.01-0.35)**	**0.13 (0-0.71)**
**Lerum**	**0.74 (0.55-0.96)**	0.61 (0.33-1.03)
**Svenljunga**	**0.47 (0.22-0.86)**	**0.13 (0-0.72)**
**Lysekil**	**0.22 (0.09-0.45)**	**0.37 (0.12-0.87)**
**Skara**	0.82 (0.55-1.18)	1.33 (0.78-2.13)
**Degerfors**	0.97 (0.59-1.5)	1.24 (0.6-2.28)
**Gagnef**	**0.5 (0.24-0.91)**	1.35 (0.65-2.49)
**Rättvik**	**0.38 (0.18-0.71)**	**0.26 (0.05-0.76)**
**Timrå**	1.33 (0.97-1.78)	1.3 (0.73-2.15)
**Dorotea**	**0 (0-0.59)**	0 (0-1.3)
**Vännäs**	**0.19 (0.04-0.54)**	**0 (0-0.62)**
**Täby**	1.09 (0.91-1.28)	1.23 (0.93-1.6)
**Strängnäs**	**0.58 (0.41-0.79)**	**0.52 (0.27-0.91)**
**Söderköping**	0.91 (0.6-1.34)	0.59 (0.22-1.28)
**Nässjö**	**1.44 (1.15-1.78)**	1.15 (0.75-1.69)
**Torsås**	0.91 (0.5-1.53)	**1.95 (1.01-3.41)**
**Västervik**	**0.33 (0.21-0.48)**	**0.43 (0.23-0.72)**
**Vellinge**	**0.25 (0.14-0.4)**	**0.16 (0.04-0.42)**
**Lomma**	**0.2 (0.09-0.38)**	**0 (0-0.23)**
**Osby**	**0.34 (0.15-0.64)**	**0.36 (0.1-0.92)**
**Tanum**	**0.43 (0.22-0.76)**	0.45 (0.15-1.05)
**Hjo**	**0.21 (0.06-0.54)**	**0 (0-0.49)**
**Ljusnarsberg**	**0.19 (0.02-0.68)**	**0 (0-0.9)**
**Karlskoga**	0.82 (0.61-1.09)	0.67 (0.38-1.09)

**Västerås**	**1.26 (1.13-1.4)**	**1.38 (1.15-1.63)**
**Köping**	1.15 (0.87-1.48)	0.84 (0.49-1.35)
**Håbo**	0.76 (0.5-1.11)	1 (0.48-1.85)
**Eskilstuna**	**1.66 (1.48-1.85)**	**1.57 (1.28-1.9)**
**Motala**	**0.64 (0.48-0.84)**	**0.39 (0.2-0.68)**
**Landskrona**	**0.25 (0.15-0.38)**	**0.04 (0-0.2)**
**Norberg**	0.67 (0.29-1.32)	0.43 (0.05-1.57)
**Arboga**	0.63 (0.37-1)	1 (0.5-1.78)
**Ragunda**	0.5 (0.18-1.09)	**0 (0-0.72)**
**Umeå**	**0.27 (0.21-0.36)**	**0.14 (0.06-0.27)**
**Älvsbyn**	**0.18 (0.04-0.52)**	**0.15 (0-0.84)**
**Eksjö**	**0.57 (0.35-0.87)**	**0.28 (0.08-0.72)**
**Klippan**	**0.21 (0.08-0.42)**	**0.16 (0.02-0.57)**
**Båstad**	**0.35 (0.18-0.61)**	**0.21 (0.04-0.61)**
**Höganäs**	**0.23 (0.13-0.4)**	**0.18 (0.05-0.47)**
**Stenungsund**	**0.47 (0.29-0.71)**	**0.13 (0.02-0.47)**
**Mark**	**0.64 (0.47-0.87)**	0.75 (0.45-1.17)
**Nordanstig**	0.67 (0.36-1.14)	0.56 (0.15-1.44)
**Kinda**	0.82 (0.48-1.31)	1.11 (0.51-2.12)
**Mullsjö**	0.51 (0.2-1.04)	0.19 (0-1.06)
**Vimmerby**	**0.22 (0.09-0.45)**	**0.24 (0.05-0.69)**
**Öckerö**	0.73 (0.44-1.15)	0.84 (0.36-1.65)
**Ale**	0.91 (0.66-1.21)	**0.26 (0.07-0.67)**
**Kumla**	1.23 (0.9-1.64)	1.07 (0.59-1.8)
**Orsa**	**0.14 (0.02-0.49)**	**0 (0-0.62)**
**Smedjebacken**	0.87 (0.53-1.34)	0.94 (0.41-1.85)
**Falun**	0.97 (0.79-1.17)	1.13 (0.82-1.52)
**Avesta**	**0.61 (0.4-0.88)**	0.68 (0.35-1.18)
**Ljusdal**	**0.63 (0.41-0.93)**	0.51 (0.22-1)
**Åsele**	**0 (0-0.58)**	0 (0-1.34)
**Haninge**	**2.13 (1.9-2.39)**	**2.6 (2.11-3.17)**
**Karlsborg**	**0.33 (0.11-0.77)**	**0 (0-0.6)**
**Hammarö**	**0.39 (0.19-0.69)**	**0.22 (0.03-0.79)**
**Kristinehamn**	**0.63 (0.43-0.89)**	**0.44 (0.2-0.84)**
**Filipstad**	1.36 (0.93-1.93)	0.93 (0.43-1.77)
**Säffle**	**0.27 (0.12-0.51)**	**0.14 (0.02-0.52)**
**Hallstahammar**	0.88 (0.58-1.27)	**0.16 (0.02-0.57)**
**Älvdalen**	**0.19 (0.04-0.57)**	**0.16 (0-0.89)**
**Säter**	0.85 (0.51-1.32)	0.72 (0.26-1.57)
**Söderhamn**	**0.52 (0.35-0.75)**	1.01 (0.62-1.54)

Significant values (95% confidence intervals) at p<0.05 are marked in bold.

Table A2. Distribution of study contextual factors in municipalities inside and outside the spatial clusters

Outcome	Variables	Municipalities inside the Clusters n=50 Median (IQR)	Municipalities outside the clusters n=240 Median (IQR)
**Hospitalizations**	Population density (Inhab./km^2)	96.1 (33.9 - 206.1)	23.1 (10.5 - 52.6)
	Gini index	0.3 (0.3 - 0.3)	0.3 (0.3 - 0.3)
	Mean income (Thousands of SEK)	316.8 (293.8 - 354.9)	293.6 (280.1 - 309.5)
	Proportion of immigrants (%)	18.7 (14.9 - 26.1)	13.4 (10.8 - 17.6)
	Proportion of inhabitants with primary education (%)	16.8 (12.8 - 20.9)	20.8 (17.6 - 23.5)
	Proportion of Population 65+ (%)	19.2 (16.1 - 24.0)	24.8 (21.7 - 27.5)
	**Variables**	**Municipalities inside the Clusters n=22 Median (IQR)**	**Municipalities outside the clusters n= 268 Median (IQR)**
**Mortality**	Population density (Inhab./km^2)	401.1 (92.9 - 1380.3)	24.8 (11.6 - 64.0)
	Gini index	0.3 (0.3 - 0.3)	0.3 (0.3 - 0.3)
	Mean income (Thousands of SEK)	358.6 (320.3 - 385.8)	294.9 (281.2 - 310.5)
	Proportion of immigrants (%)	20.5 (17.6 - 29.3)	13.7 (10.9 - 17.8)
	Proportion of inhabitants with primary education (%)	12.8 (10.2 - 14.8)	20.7 (17.4 - 23.2)
	Proportion of Population 65+ (%)	16.6 (15.4 - 19.3)	24.6 (21.3 - 27.2)

Table A3. Rate ratio of hospitalizations and mortality between population younger than 65 and aged 65+ within spatial clusters and outside spatial clusters.

Outcome		Municipalities outside the clusters	Municipalities inside the Clusters
**Hospitalizations**	Hospitalizations 65+	3706	5762
	Population 65+	1363551	701816
	Hospitalizations <65	3176	5603
	Popopulation <65	4858684	3403538
	Rate Ratio (95%CI)	4.16 (3.97 - 4.36)	4.99 (4.81 - 5.17)
	p-value	<0.001	<0.001
**Mortality**	Deaths 65+	3403	2600
	Population 65+	1654323	411044
	Deaths <65	186	195
	Popopulation <65	6055915	2206307
	Rate Ratio (95%CI)	66.97 (57.78 - 77.63)	71.57 (61.88 - 82.78)
	p-value	<0.001	<0.001
